# An Improved Approach to Automated Measurement of Body Condition Score in Dairy Cows Using a Three-Dimensional Camera System

**DOI:** 10.3390/ani12010072

**Published:** 2021-12-29

**Authors:** Rodrigo I. Albornoz, Khageswor Giri, Murray C. Hannah, William J. Wales

**Affiliations:** 1 Agriculture Victoria Research, Ellinbank, VIC 3821, Australia; murray.hannah@agriculture.vic.gov.au (M.C.H.); bill.wales@agriculture.vic.gov.au (W.J.W.); 2 Agriculture Victoria Research, Bundoora, VIC 3083, Australia; khageswor.giri@agriculture.vic.gov.au; 3 Centre for Agricultural Innovation, School of Agriculture and Food, Faculty of Veterinary and Agricultural Sciences, The University of Melbourne, Melbourne, VIC 3010, Australia

**Keywords:** body condition score, 3D camera, sensitivity, animal research, automation

## Abstract

**Simple Summary:**

Body condition scoring is a valuable tool used to assess the changes in subcutaneous body tissue reserves of dairy cows throughout the lactation. A visual method is typically used to assign a body condition score (BCS) to a cow following a standardized scale based on an assessment of tissue cover in the hind quarters of the animal. This method is subject to operator bias and is labor intensive, limiting the number of animals that can be scored and frequency of measurement. The objective of this study was to evaluate the suitability of an automated 3D body condition scoring camera system with capability to measure BCS daily as an alternative to visual body condition scoring for research applications. We found that the camera system using raw data greatly increased precision and ability to detect changes in BCS within dairy cows over time compared with the visual method assessed weekly. For research applications, the precision and sensitivity were further improved by a proposed refinement of the camera’s daily BCS data.

**Abstract:**

Body condition scoring is a valuable tool used to assess the changes in subcutaneous tissue reserves of dairy cows throughout the lactation resulting from changes to management or nutritional interventions. A subjective visual method is typically used to assign a body condition score (BCS) to a cow following a standardized scale, but this method is subject to operator bias and is labor intensive, limiting the number of animals that can be scored and frequency of measurement. An automated three-dimensional body condition scoring camera system is commercially available (DeLaval Body Condition Scoring, BCS DeLaval International AB, Tumba, Sweden), but the reliability of the BCS data for research applications is still unknown, as the system’s sensitivity to change in BCS over time within cows has yet to be investigated. The objective of this study was to evaluate the suitability of an automated body condition scoring system for dairy cows for research applications as an alternative to visual body condition scoring. Thirty-two multiparous Holstein-Friesian cows (9 ± 6.8 days in milk) were body condition scored visually by three trained staff weekly and automatically twice each day by the camera for at least 7 consecutive weeks. Measurements were performed in early lactation, when the greatest differences in BCS of a cow over the lactation are normally present, and changes in BCS occur rapidly compared with later stages, allowing for detectable changes in a short timeframe by each method. Two data sets were obtained from the automatic body condition scoring camera: (1) raw daily BCS camera values and (2) a refined data set obtained from the raw daily BCS camera data by fitting a robust smooth loess function to identify and remove outliers. Agreement, precision, and sensitivity properties of the three data sets (visual, raw, and refined camera BCS) were compared in terms of the weekly average for each cow. Sensitivity was estimated as the ratio of response to precision, providing an objective performance criterion for independent comparison of methods. The camera body condition scoring method, using raw or refined camera data, performed better on this criterion compared with the visual method. Sensitivities of the raw BCS camera method, the refined BCS camera method, and the visual BCS method for changes in weekly mean score were 3.6, 6.2, and 1.7, respectively. To detect a change in BCS of an animal, assuming a decline of about 0.2 BCS (1–8 scale) per month, as was observed on average in this experiment, it would take around 44 days with the visual method, 21 days with the raw camera method, or 12 days with the refined camera method. This represents an increased capacity of both camera methods to detect changes in BCS over time compared with the visual method, which improved further when raw camera data were refined as per our proposed method. We recommend the use of the proposed refinement of the camera’s daily BCS data for research applications.

## 1. Introduction

Body condition of dairy cows is an indirect indicator of their subcutaneous body reserves status. Depletion of body reserves in dairy cows normally occurs in early lactation, and accretion occurs towards the end of the lactation [1]. Research has shown that over-fat or -skinny cows at calving or rapid loss of body condition in early lactation can predispose cows to lower milk production, negative health outcomes, and poorer reproductive performance [2,3]. Further, excessive loss of body condition in early lactation has been associated with decreased survival of cows in the herd [4]. Therefore, assessing and managing the rate of change in body condition and achieving optimum body condition at different stages of lactation are key factors for maintaining or improving performance and welfare of dairy cows.

Assessment of cow body condition is typically performed by at least two operators utilizing one of the available body condition score (BCS) scales [5,6,7] that are linearly related with each other [8]. Visual body condition scoring consists of a visual evaluation of anatomical points from the rear-end of the dairy cow (e.g., hooks and tail-head area), where changes in subcutaneous body reserves are visually more evident, to assign a score according to a standardized scale. However, this technique includes operator subjectivity as well as requiring the animal to be restrained in some cases during the evaluation process. This is a labor-intensive technique that limits the number or frequency of animals that can be scored. Therefore, implementation of an automated body condition scoring system has the potential to remove subjectivity in scoring, minimize stress to the animal, and increase the number and frequency of measurements collected.

Automated sensor technologies are increasingly being researched and developed for precision livestock farming [9]. In recent years, an automatic three-dimensional (3D) body condition scoring camera system mounted over areas of normal cow traffic flow has become commercially available in recent years (DeLaval Body Condition Scoring, BCS DeLaval International AB, Tumba, Sweden). This camera system individually identifies cows fitted with transponders via a radio-frequency identification reader and allows for multiple BCS measurements within a day, with the camera software recording individual daily BCS values for each scoring session and reporting a daily trimmed, 7-d rolling average of BCS. The rolling average is derived from measurements made over the preceding seven days, with the lowest and highest 20% of values removed as outliers. A recent study reported a strong positive correlation (0.78) between BCS values obtained visually and those reported by the same camera system, with the camera system reporting agreement with visual BCS values within a range commonly observed in dairy cows (3–3.75 using a 1–5 scale) but disagreement for cows outside of that BCS range [10]. In that study, authors suggested that the inaccuracy of the automated system may be related to failure of the system’s algorithm to capture key differences in physical features of under- and over-conditioned cows, and therefore, efforts to improve the system’s algorithms could enhance its reliability. On the other hand, lack of agreement between camera and visual BCS could be due to unreliability of visual scoring that is not at a proper interval scale. This question is particularly important for research applications, which require a precise measure for reporting in the literature rather than a comparative ranking that might be satisfactory for commercial dairy farming purposes.

Mullins et al. [10] compared visual BCS observations from 343 dairy cows against individual daily 7-d rolling averages estimated by the camera software. Furthermore, Mullins et al. [10] used correlation coefficients to study the linear relationship and Bland–Altman plot to study the agreement between camera scores and visual scores, but no measure of objective performance for each method was reported.

Our objective was to evaluate the suitability of a commercially available, automatic, 3D body condition scoring camera system for dairy cows as an alternative to visual body condition scoring, firstly by investigating agreement between visual and camera BCS and, secondly, by comparing the performance of the two measurement methods in terms of their relative sensitivity as a measure of objective performance [11]. The automatic 3D camera system has obvious practical advantages. We hypothesize that it will also be more sensitive for measuring body condition compared to a visual body condition scoring method.

## 2. Materials and Methods

This study was conducted in accordance with the Australian Code of Practice for the Care and Use of Animals for Scientific Purposes (National Health and Medical Research Council, 2004). Animal use was approved by the Animal Ethics Committee of the Department of Jobs, Precincts, and Regions, Victoria (AEC 2019-09). Thirty-two multiparous Holstein-Friesian cows in early lactation (9 ± 6.8 days in milk and 4.8 ± 1.30 years old) were visually body condition scored on a weekly basis on the same day of the week and automatically, twice daily, from 1 August to 30 September 2019. This study was conducted in early lactation, when the highest and lowest BCS values in the lactation are typically observed, and changes in BCS occur rapidly compared with later stages. The frequency of visual measurements in this study was justified by the rapid and detectable changes in BCS that occur during the first 7–10 weeks after calving. Measurements were performed for at least 7 consecutive weeks for all cows, except for one cow that exited the experiment early due to locomotive problems and was only scored over 3 consecutive weeks. Cows were fed a grain mix concentrate twice daily at each milking (approximately 06:15 and 15:15 h) throughout the study, offered pasture silage while housed in a barn facility during the first three weeks after calving, and subsequently held outdoors and offered perennial ryegrass pasture for the remainder of the measurement period. Visual BCS measurements were performed independently on each cow after the morning milking by three trained staff throughout the study using the 1–8 BCS scale with 0.25-point increments [6]. Automatic BCS measurements were recorded after each milking using a commercially available 3D body condition scoring camera systems (DeLaval Body Condition Scoring, BCS DeLaval International AB, Tumba, Sweden) with two cameras, each mounted over one of the milking parlor exit races. Cows were individually identified via a radio-frequency identification collar system allowing for multiple BCS measurements in a day. Hence, each cow typically had 3 visual BCS measurements performed during the same day each week and 14 automatic BCS measurements per week. The camera system reports BCS values in increments of 0.1 points and offers two scale settings, 1–5 scale [5] or 1–10 scale [7], and for the purpose of this study, the system was set to the 1–10 BCS scale. This scale was converted to the 1–8 scale [6] by applying the published linear equation of Roche et al. [8]. Data from the camera system are reported as 7-d BCS rolling average (not used for the purpose of this study) that, prior to averaging, removes the lowest and highest 20% of values or as daily AM and PM BCS values. Individual daily AM and PM raw BCS data from each of the cameras was accessed via the manufacturer’s software (DelPro Farm Manager, DeLaval International AB, Tumba, Sweden) using the pathway Systems>Devices>BCS Camera>BCS CAM, as data are not readily available for download and were downloaded weekly before data were automatically overwritten by the system after 8 days.

### Statistical Analyses

The raw camera data were initially examined graphically for errors. About 8% of the camera BCS data were either zero or negative, which were treated as erroneous and excluded from further analyses. For the raw camera method, analyses were performed on data with zero and negative values removed but including other outlier points. For the refined camera method, data identified as outliers were also removed after allowing for effects of cow and time using a robust smoothing function, loess [12]. This smoother was fitted to the camera BCS time series data for each cow using R software [13], with loess arguments, span = 0.5, and family = “symmetric.” The “symmetric” setting selects an iterative algorithm that down-weights the effect of outliers on the fitted smooth line. Outliers were identified using Tukey’s rule [14] as any value having a residual greater than 1.5 times the interquartile range, either below the first quartile or above the third quartile of the distribution of residuals. This method flexibly allows for trends in body condition with time, removing outliers objectively identified, and so differs from the method used in the camera system to obtain the trimmed 7-d BCS rolling average that, prior to averaging, removes the lowest and highest 20% of values.

The Pearson correlation coefficient (CC) and Lin’s concordance correlation coefficient (LCCC) [15] were calculated to measure linear relationship and agreement between camera and visual methods, using animal by week mean data. Agreement was also analysed using Bland–Altman plots of difference versus mean of the two methods, using animal by week mean data and entailed calculation of the mean difference, the standard deviation of difference, and 95% range for the difference [16].

Sensitivity (denoted by Θ in this paper), as proposed by Mandel and Stiehler [11], modified by Hannah et al. [17], and defined as

(1) $$\Theta =\frac{\sigma_{a}}{\sigma_{m}},$$

where the numerator, $\sigma_{a}$, represents actual standard deviation in body condition, and the denominator, $\sigma_{m}$, is the measurement error standard deviation (i.e., standard error), was calculated for each measurement method. In each case, the standard deviations, $\sigma_{a}$ and $\sigma_{m}$, related to a cow by week, mean were calculated, to ensure fair comparison of the measurement methods at a common temporal and physical scale. These standard deviations were themselves calculated from components of variance as described below, derived under each sampling design and estimated in detailed random-effects models [18]. The random-effects models included all available terms in the respective sampling structures, as listed in Table 1, and were fitted separately to the camera and visual BCS data using REML software in Genstat 20 (VSNi, 2019).

Estimates of $\sigma_{a}$ and $\sigma_{m}$ were calculated differently for the detection of changes in body condition over time within animals and for the detection of differences between animals at a given time. These involved different but overlapping sets of variance components. In the following, the subscripts *W, D, M, A, C, S* and *ε*, of variance components, $\sigma^{2}$, refer to week, day, milking, animal, camera, scorer and residual error, respectively. For changes over time, $\sigma_{a}^{2}=\sigma_{W}^{2}+\sigma_{WA}^{2}$, and for differences between animals, $\sigma_{a}^{2}=\sigma_{A}^{2}+\sigma_{WA}^{2}$, where $\sigma_{W}^{2}$, $\sigma_{A}^{2}$, and $\sigma_{WA}^{2}$ were variance components for week, animal, and animal by week, respectively. These equations were applied for each measurement method.

The error variances denoted by $\sigma_{m}^{2}$, were calculated as

(2) $$\sigma_{m}^{2}=\underset{i\in M}{\sum }\frac{\sigma_{i}^{2}}{n_{i}},$$

where *M* was the set of indices of relevant variance components that contributed to error, and $n_{i}$ was the number of effects from each factor that were averaged into an animal by week mean. In the case of visual BCS, Equation (2) is expanded to

(3a) $$\sigma_{m}^{2}=\frac{\sigma_{WS}^{2}}{3}+\frac{\sigma_{\varepsilon }^{2}}{3},$$

for changes with time, or

(3b) $$\sigma_{m}^{2}=\frac{\sigma_{AS}^{2}}{3}+\frac{\sigma_{\varepsilon }^{2}}{3},$$

for differences between animals, with the components defined as per Table 1. The denominators, $n_{i}$ = 3, derive from there being 3 scores (once per week by 3 scorers) for each animal. Note that the scorer variance component, $\sigma_{S}$^2^, does not appear as part of this effective error variance. This was because, under the sampling design of this study, scorer main effects were orthogonal to animal by week and thus not involved in comparisons over weeks nor between animals.

In the case of the camera BCS, most of the variance components not included in $\sigma_{a}^{2}$ were included in $\sigma_{m}^{2}$; thus, Equation (2) expanded to

(4a) $$\sigma_{m}^{2}=\frac{\sigma_{WD}^{2}}{7}+\frac{\sigma_{WC}^{2}}{2}+\frac{\sigma_{WDM}^{2}}{14}+\frac{\sigma_{WDA}^{2}}{7}+\frac{\sigma_{WDC}^{2}}{14}+\frac{\sigma_{WAC}^{2}}{2}+\frac{\sigma_{WDMA}^{2}}{14}+\frac{\sigma_{WDMC}^{2}}{14}+\frac{\sigma_{WDAC}^{2}}{14}\;+\frac{\sigma_{WDMAC}^{2}}{14}+\frac{\sigma_{\varepsilon }^{2}}{14},$$

for changes with time, or

(4b) $$\sigma_{m}^{2}=\frac{\sigma_{AC}^{2}}{2}+\frac{\sigma_{WDA}^{2}}{7}+\frac{\sigma_{WAC}^{2}}{2}+\frac{\sigma_{WDMA}^{2}}{14}+\frac{\sigma_{WDAC}^{2}}{14}+\frac{\sigma_{WDMAC}^{2}}{14}+\frac{\sigma_{\varepsilon }^{2}}{14},$$

for differences between animals within time, with the variance components defined in Table 1. The main effect of camera, while not orthogonal to animal and week effects (since animals self-selected camera after each milking), could nonetheless be accounted for as a block effect and so was not included in these formulae. If camera devices operated consistently independent of animal or time, many of these variance components could be expected to be zero.

The sensitivity of each camera BCS measurement method relative to the visual BCS measurement method was calculated as a relative sensitivity (8):

(5) $$RS=\frac{\Theta_{cam}}{\Theta_{vis}}$$

where $\Theta_{cam}$ and $\Theta_{vis}$ are sensitivity for camera and visual BCS measurement method respectively. Using this ratio, it is possible to calculate a sampling intensity (i.e., the number of independent measurements per animal by week mean) under one method that would be needed for its sensitivity to equal that of the other method. For example, the error variance $\sigma_{m}^{2}$ of a visual BCS of a cow by week mean is inversely proportion to the number of samplers (Equation (3a,b)). Consequently, *RS* (Equation (5)), via Equation (1), is proportional to the square-root of the number of samplers since $\sigma_{a}^{2}$ is independent of this number. It follows that the number of samplers needed to render the sensitivity of the visual BCS to equal that of the camera is $RS^{2}$ times the current number of visual samplers. This was calculated for both the raw and refined camera methods.

An average rate of change in BCS per month was estimated from our data as the slope, β, of a linear regression of BCS against time. A detectable change in body condition would be, say, $3\sigma_{m}$, for a single animal. Hence, the time required for a change in body condition to be detected was calculated as

(6) $$t=3\sigma_{m}/\beta$$

under each method.

## 3. Results

**Figure 1:**
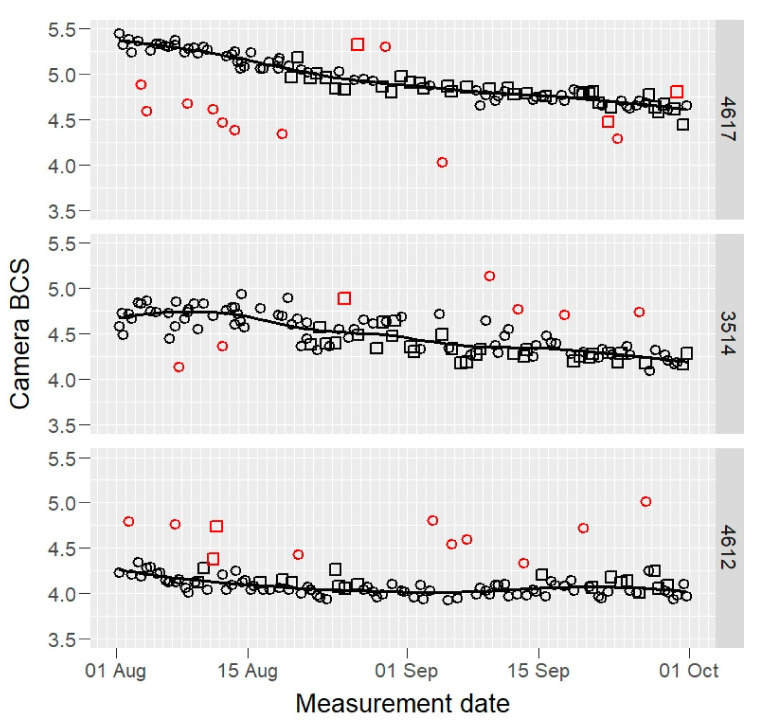
Raw camera body condition score (BCS) values of three selected cows (4617, 3514, and 4612: maximum, median, and minimum BCS mean value cows) by two cameras (open squares and circles) during the experiment, on 1–8 scale. The fitted solid line is a robust loess smooth curve that was used to identify the outliers (red open squares and circles).

**Figure 2:**
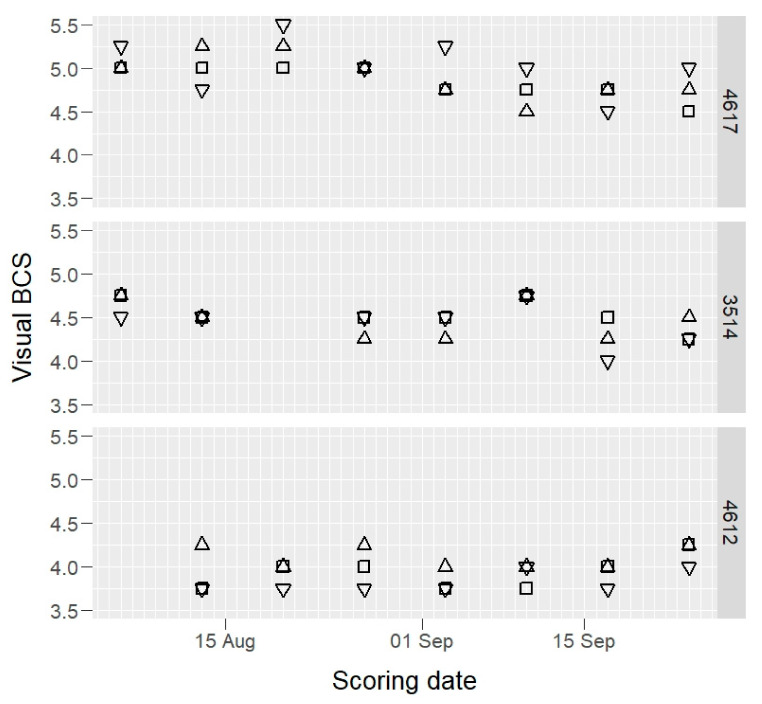
Visual body condition score (BCS) values of three selected cows (4617, 3514, and 4612: maximum, median, and minimum BCS mean value cows) by three scorers (open squares, open triangles, and inverted open triangles) on 1–8 scale.

Raw BCS data of three example cows, those having maximum, median, and minimum mean BCS values, are presented in Figure 1 (camera) and Figure 2 (visual).

**Figure 3:**
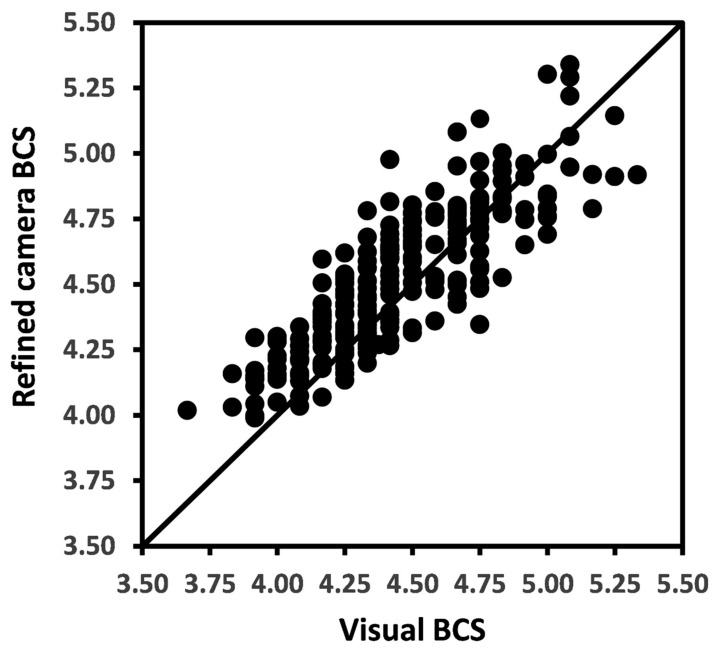
Scatter plot with each solid dot representing a weekly mean body condition score (BCS) for a cow by refined camera method versus visual measurement method. The solid line represents the line of agreement.

The overall BCS means for raw camera, refined camera, and for visual methods were 4.50, 4.49, and 4.44 (Table 1). The Pearson’s CC and LCCC between raw camera method and visual methods of BCS measurements were 0.85 and 0.81 and between refined camera and visual methods were 0.86 and 0.83, respectively. The relationship between refined camera BCS and visual BCS is presented in Figure 3.

**Figure 4:**
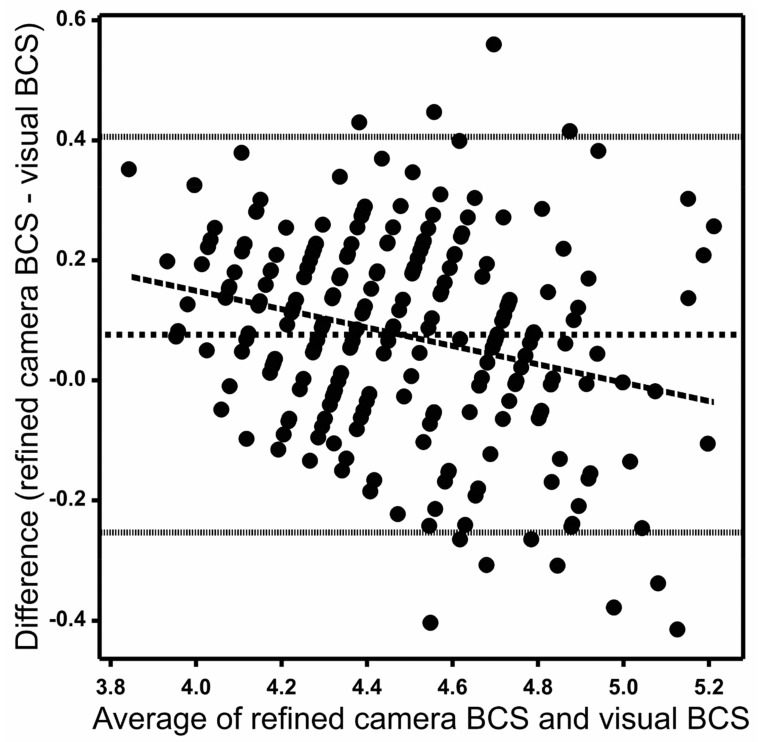
Bland–Altman plot with each solid dot representing a weekly mean body condition score (BCS) for a cow between refined camera and visual measurement methods. The central horizontal dotted line represents the mean difference between the two measurement methods, and the fine dotted lines represent the 95% range of the differences. The long-dashed line is a linear regression (*p* < 0.05) of difference versus the average.

Figure 4 shows the Bland–Altman (difference vs. average) plots of weekly animal means for the refined camera data and the visual method. The plot shows a mean difference of 0.07 between the refined camera and the visual method and 95% of differences between −0.26 and 0.41 on the y-axis. The standard deviation of differences between the refined camera and the visual method was 0.16. The differences between the camera and the visual method trended downwards with increasing average, with the differences being mostly positive (camera value greater than visual score) for smaller mean values and mostly negative (camera value less than visual score) for larger mean BCS values (Figure 4).

The diagonally striped pattern of points visible in the graph is an artefact of the discreetness of the 1–8 visual score scale, scored to the nearest 0.25. The average score from three scorers therefore allowed 12 possible distinct values in increments of 0.083 per unit score. These manifested as 12 diagonal rows of points between 4.0 and 5.0 on the x-axis on Figure 4. The discreteness of the visual scoring scale could therefore account for only about one-tenth of the differences actually observed between camera and visual scores.

The scatter plot and the Bland–Altman plot for raw camera data with visual BCS are not shown. These were very similar to those for the refined data in Figure 3 and Figure 4. The mean difference between the raw camera and the visual method was 0.08. The standard deviation of differences between the raw camera and the visual method was 0.17. The exclusion of outliers in the refined data had little visual effect on the figures since each point on these graphs represents an animal by week average of 14 camera data, of which only one on average was an outlier.

The variance components associated with random-effect terms for camera and visual BCS and their estimates are provided in Table 1. Most of the variance measured by each method was explained by animal, week, and their interaction. These three correspond to actual differences in body condition. The largest component was the variance between animals, followed by between weeks, and lastly animal by week (Table 1). Of the components contributing to error, the largest was the residual variance in each case.

The effective error standard deviation for an animal by week mean relevant to changes over time (Equations (3a) and (4a)) was 0.041, 0.026, and 0.083 for raw camera, refined camera, and visual methods, respectively (Table 2). The sensitivity to change in cow BCS between weeks was greatest for the refined camera method (6.2), intermediate for the raw method (3.6), and least for the visual method (1.7, Table 2). The relative sensitivity, *RS*, was 3.7 for the refined camera method and 2.1 for the raw camera method relative to the visual method. The square of these *RS* indicates that 14 times as many scorers (that is, 3.7^2^ × 3 = 41 scorers in this experiment) would have been needed for the visual scoring method to match the performance of the refined camera method in terms of its sensitivity and about five times as many scorers (i.e., 2.1^2^ × 3 = 14 scorers) to match the performance of the raw camera method. The estimated rate of change, β, in BCS observed in this experiment is shown in Table 2. It would take 44 days to detect a change in BCS in an animal using visual BCS method but 21 or 12 days to detect a change using the raw or refined camera methods, respectively (Table 2).

Standard errors for detecting differences between animals within week (Equations (3b) and (4b)) were 0.048, 0.030, and 0.090 of a BCS for raw camera, refined camera, and visual methods, respectively (Table 2). The corresponding sensitivities were 4.3, 7.8, and 3.2 (Table 2). The relative sensitivities were 1.3 and 2.4 for the raw and refined camera methods relative to the visual method. The squares of these relative sensitivities indicate that six times as many scorers would be needed for the visual scoring method to match the performance of the refined camera method, and two times as many scorers would be needed to match the performance of the raw camera method.

## 4. Discussion

Body condition score and its change over time in dairy cows are valuable metrics for evaluation of management and nutritional programs in production or research settings given their associations with cows’ production, health, reproduction, and welfare traits [2,3,4]. The visual method used to evaluate BCS of dairy cows has been questioned not only because of the subjectivity associated with a visual assessment and interpretation of that information to assign a BCS value [19] but also because it is deemed, by default, as the traditional standard for comparison with other methodologies [10]. The findings of this research indicate a more objective technology-driven methodology represents a better reference method in commercial and research settings.

Body condition score measurements were performed between calving or very early postpartum and weeks 7 or 10 after calving. This allowed for individual measurements of BCS when cows normally record their highest and lowest BCS values and the greatest changes in BCS throughout the lactation in a short timeframe. The statistical metrics employed in this study demonstrated a high degree of correlation and agreement between the camera and visual BCS. The Pearson CC of 0.86 and LCCC of 0.83 between the refined camera and visual methods, calculated on animal by weekly means, while below unity, were high given the restricted range of visual BCS data from 3.6 to 5.4 compared with the full 1–8 scoring scale. Correlation coefficients depend on data range as well as linear relationship [16], and a narrow range of data results in a smaller estimate of correlation. On the other hand, as correlations are between means, they would be larger than correlations between individual measurements. Nonetheless, these results were consistent with observations by Mullins et al. [10], who, in a Bland–Altman plot, showed agreement between BCS values obtained with the same camera system as was used in our study but who used the trimmed 7-d BCS rolling average data readily available and reported by the system’s software and a visual method using the 1–5 BCS scale [5].

Some small systematic differences between visual and camera scores were observed in our data, with the mean camera BCS being 0.08 greater than the mean visual BCS. There was also a tendency for this difference to be positive when body condition was low and for it to be negative when the body condition was high. This trend, albeit small and accounting for at most 0.1 of a BCS unit at the extremes of our data (Figure 4), could have arisen by a small calibration error either within the camera software to its 1–10 scale or between the 1–10 and the 1–8 scale. It could equally be due to inaccuracy in visual scoring. Alternatively, it may be an artefact of unequal error variances in the two measurement variables, in this case visual BCS and refined camera BCS, in the Bland–Altman plot as outlined by Francq and Govaerts [20]. An underestimate of calibration slope would lead to under-estimates of camera values at large BCS and over-estimates at small BCS, as was apparent in Figure 3 and Figure 4. This flattening of calibration relationship would also lead to under-estimation of standard deviations $\sigma_{a}$ and $\sigma_{m}$ for the camera methods.

An accurate calibration of the camera to visual BCS scale is important for continuity of interpretation of animal management standards already in place and expressed in terms of the visual 1–8 BCS scale. While recalibration could be achieved by regressing our visual score data on our camera data, and doing so would increase method agreement marginally within this study, recalibration would not be an option available to studies that did not employ both measurement methods simultaneously. Apart from this impracticality, calibration depends for its success on the quantity, quality, and range of data at hand. Repeated recalibration across studies would compromise consistency of the camera method, which otherwise is among its main advantages over a manual method. The calibration supplied with the camera in conjunction with that of Roche et al. [8] appears to be adequate to preserve interpretation of the standard 1–8 BCS scale, and no recalibration is recommended.

To be effective, a measurement method must be both responsive and precise. A method that fails to be responsive, for example, by returning a constant BCS of “4”, would be useless despite its perfect (zero-variance) precision. The statistic for sensitivity defined in Equation (1) offers a criterion by which performance of the measurement methods can be compared. It quantifies response and precision simultaneously, reflecting the capability of a measurement method to detect real effects. Sensitivity, so defined, is invariant to linear changes of scale so that it remains unchanged whether the camera BCS is expressed on a 1–5, a 1–8, or a 1–10 scale or whether the linear calibration is not quite right. It does so by giving the response on the scale as a proportion of precision on the same scale. The choice of scale and its calibration, then, are a separate issue. The relative sensitivity (*RS*, Equation (5)) facilitates comparison of two methods in a way that is also independent of the magnitude of the biological effects present in the experiment, provided the responses in the two measures remain linearly related to those effects. The *RS* provides a relevant and reliable criterion by which the essential performance of two methods can be compared [11].

In the current experiment, comparisons were made at the scale of an animal by week mean, with camera data accumulated twice daily and visual BCS performed by three scorers once each week. The sensitivity of the refined camera method was 3.7 (i.e., the *RS*) times that of the sensitivity of the visual method of body condition scoring for detecting changes. This represents a considerable improvement in performance of the refined camera method over the visual method. The sampling intensity of the visual BCS would have to be increased 14 (i.e., *RS*^2^)-fold, from 3 to 41 independent visual scorers, to equal the performance of the refined camera. Such was its sensitivity that the refined camera could detect a change in BCS in an estimated 12 days rather than the 44 days for the visual method (Table 2) given the average rate of change observed and the sampling protocols in our experiment. Without excluding outliers, the raw camera method would take an estimated 21 days to detect a change in BCS, and there would need to be *RS*^2^ ≈ 5 times as many visual scorers to match its sensitivity. Substantial gains in performance were also observed for detecting differences between animals within a week, with *RS* values of 2.4 for the refined camera method and 1.3 for the raw camera method relative to the visual method. These represent an increased capability of both camera methods for detecting changes and differences in BCS compared with the visual method but especially when raw data were refined as per our statistical method for filtering outliers.

A single BCS measurement from the refined camera data had greater precision and smaller residual standard deviation, ($\sigma_{\varepsilon }$ = 0.08) compared with a single BCS measurement from the camera system raw data ($\sigma_{\varepsilon }$ = 0.15) or a single score from the visual method ($\sigma_{\varepsilon }$ = 0.14; Table 1). The single-measurement gain in precision for the refined camera was modest and non-existent for the raw camera data over the visual method. The substantial gains in sensitivity observed were therefore largely a consequence of the greater measurement frequency and hence a greater number of measurements per cow per week made by the camera system (typically 14 measurements in total from twice a day measurements) compared with visual scoring (three measurements on just one day of the week). The increased precision and sensitivity offered by the refined camera method make it the preferred method, especially for research applications, particularly when an experiment is enrolling a limited number of animals, based on the principles of refinement and reduction for the ethical and economic use of animals in research.

To our knowledge, only one study has reported the ability of a 3D camera system to detect changes on specific points of the dorsal portion of the animal (e.g., rump angle, pin width) over time within cows [21]. However, in that study, the ability of the camera system to detect changes over time was interpreted based on agreement between the camera and visual measurements. The 7-d rolling BCS trimmed average provided by the camera software is similar in concept to our loess-filtered 7-d mean. The loess-filtered version should perform a little better, as it allows for time trend and discards fewer data, only excluding data when their distance from the center of their distribution is sufficient to identify them as outliers, which turned out to be around 7% of the data.

The BCS means obtained by all three methods compared in our study ranged from 3.6 to 5.4 and were mostly within the recommended range for cows in early lactation (4.5–5 points in a 1–8 scale). How the camera method behaves outside this range remains to be tested; however, cows’ BCS is not typically observed and managed outside of this range, as it predisposes them to decreased productive and health performance and to animal welfare concerns in extreme cases in both research and commercial farms.

## 5. Conclusions

The automated 3D camera system used in this study (DeLaval Body Condition Scoring, BCS DeLaval International AB, Tumba, Sweden) is a suitable option for measuring BCS in dairy cows in research and commercial dairy farming situations. The camera system greatly increased precision and ability to detect differences between animals and changes in BCS within animals over time compared with the traditional visual method. Further, improved precision and sensitivity was achieved by a proposed refinement of the camera’s raw BCS data by fitting a robust loess smoother with removal of outliers.

## Figures and Tables

**Table 1:** Estimates of random-effect model parameters (mean and variance components) for each of raw camera, refined camera, and visual body condition score (BCS) data. Camera measurements were taken at least twice daily for a period of 7 weeks on each of 32 cows. The same cows were scored visually for BCS by three scorers on one day of each week over the same period.

Model Parameters	Raw Camera	Refined Camera	Visual Scoring
Mean BCS	4.50	4.49	4.44
Variance components (×10^−2^):			
Week ($\sigma_{W}^{2}$)	1.70	1.90	1.00
Animal ($\sigma_{A}^{2}$)	3.75	4.63	7.36
Camera ($\sigma_{C}^{2}$), or Scorer $(\sigma_{S}^{2}$)	0.00	0.00	0.04
Week.Day $\left(\sigma_{WD}^{2}\right)$	0.05	0.04	
Week.Animal ($\sigma_{WA}^{2})$	0.52	0.77	0.94
Week.Camera ($\sigma_{WC}^{2}$), or Week.Scorer ($\sigma_{WS}^{2}$)	0.00	0.00	0.22
Animal.Camera ($\sigma_{AC}^{2}$), or Animal.Scorer ($\sigma_{AS}^{2}$)	0.14	0.06	0.56
Week.Day.Milking ($\sigma_{WDM}^{2})$	0.00	0.00	
Week.Day.Animal ($\sigma_{WDA}^{2})$	0.00	0.06	
Week.Day.Camera ($\sigma_{WDC}^{2})$	0.00	0.00	
Week.Animal.Camera ($\sigma_{WAC}^{2})$	0.00	0.00	
Week.Day.Milking.Animal ($\sigma_{WDMA}^{2})$	0.00	0.12	
Week.Day.Milking.Camera($\sigma_{WDMC}^{2})$	0.02	0.03	
Week.Day.Animal.Camera ($\sigma_{WDAC}^{2})$	0.092	0.00	
Week.Day.Milking.Animal.Camera ($\sigma_{WDMAC}^{2})$	0.00	0.00	
Residual ($\sigma_{\varepsilon }^{2})$	2.18	0.62	1.86

Subscripts *W*, *D*, *M*, *A*, *C*, *S* and *ε*, of variance components, $\sigma^{2}$, refer to week, day, milking, animal, camera, scorer and residual error, respectively.

**Table 2:** Estimates of summary statistics for comparison of raw camera, refined camera, and visual body condition score (BCS) measurement methods. Each statistic relates to the response and/or precision of a cow by week mean. Under both camera methods each mean was an average of approximately 14 camera measurements, and under the visual scoring method, each mean was an average of 3 independent scores.

Summary Statistics	Raw Camera	Refined Camera	Visual Scoring
For change within animal over time:			
Actual SD, $\sigma_{a}$, (BCS)	0.149	0.164	0.139
Error SD, $\sigma_{m}$, (BCS)	0.041	0.026	0.083
Sensitivity, Θ	3.6	6.2	1.7
Relative Sensitivity, *RS*, of camera to visual scoring	2.1	3.7	
Rate of change in BCS, *β*, (BCS/Month)	−0.18	−0.19	−0.17
Time to detect BCS change of $3\sigma_{m}$, (Days)	21	12	44
For differences between animals at the same week:			
Actual SD, $\sigma_{a}$, (BCS)	0.207	0.232	0.288
Error SD, $\sigma_{m}$, (BCS)	0.048	0.030	0.090
Sensitivity $\left(\Theta =\sigma_{a}/\sigma_{m}\right)$	4.3	7.8	3.2
Relative Sensitivity, *RS*, of camera to visual scoring	1.3	2.4	

SD—Standard deviation.

## Data Availability

The data presented in this study are available on request from the corresponding author.
